# Mapping the Association of College and Research Libraries information literacy framework and nursing professional standards onto an assessment rubric

**DOI:** 10.5195/jmla.2017.39

**Published:** 2017-04

**Authors:** Gloria Willson, Katelyn Angell

## Abstract

**Objective:**

The authors developed a rubric for assessing undergraduate nursing research papers for information literacy skills critical to their development as researchers and health professionals.

**Methods:**

We developed a rubric mapping six American Nurses Association professional standards onto six related concepts of the Association of College & Research Libraries (ACRL) Framework for Information Literacy for Higher Education. We used this rubric to evaluate fifty student research papers and assess inter-rater reliability.

**Results:**

Students tended to score highest on the “Information Has Value” dimension and lowest on the “Scholarship as Conversation” dimension. However, we found a discrepancy between the grading patterns of the two investigators, with inter-rater reliability being “fair” or “poor” for all six rubric dimensions.

**Conclusions:**

The development of a rubric that dually assesses information literacy skills and maps relevant disciplinary competencies holds potential. This study offers a template for a rubric inspired by the ACRL Framework and outside professional standards. However, the overall low inter-rater reliability demands further calibration of the rubric. Following additional norming, this rubric can be used to help students identify the key information literacy competencies that they need in order to succeed as college students and future nurses. These skills include developing an authoritative voice, determining the scope of their information needs, and understanding the ramifications of their information choices.

## INTRODUCTION

In much of the literature about information literacy (IL) instruction for nursing students, educational interventions have focused on teaching students how to search the library’s resources. What may be missing from most IL instruction is providing context or the reason why the information that they seek is necessary and important, such as for making informed clinical decisions (i.e., evidence-based health care). Health librarianship literature suggests weak ties to the overall concept of IL from the library profession, but there is a considerable amount of nursing literature on the topic [1]. This suggests that academic health sciences librarians do not tend to utilize established IL frameworks or apply them in planning their IL instruction as much as general academic librarians do. Nursing faculty, however, see the importance of IL, but interdisciplinary connections or collaborations between information science and nursing may not be frequent. Kuglitsch concludes that library scholars have paid little attention to transfer of knowledge and skills between disciplines, even though it is one of the most important features of IL transfer in the university [2].

A medium-sized university with undergraduate and graduate programs in the field of nursing, Brooklyn Campus, Long Island University, is highly committed to educating nurses. Undergraduates receive a bachelor’s of science in nursing, and graduates choose from a master’s of science as adult nurse practitioner, family nurse practitioner, or nurse educator. The university seeks to teach nursing students the diverse range of IL competencies that are required for them to flourish as students and nursing professionals. The authors’ definition of IL is that outlined in the Association of College & Research Libraries (ACRL) Framework for Information Literacy for Higher Education (Framework), which describes IL as a “set of integrated abilities encompassing the reflective discovery of information, the understanding of how information is produced and valued, and the use of information in creating new knowledge and participating ethically in communities of learning” [3].

One of the core requirements of undergraduate nursing majors at our university is a writing-intensive course called “End of Life Care” (EOLC). This course teaches students fundamentals of reflection, critical thinking, research, and communication integral to serving this sensitive patient population. The major course project is a six-page patient/intervention/comparison/outcome (PICO) research paper that directs students to devise a solid clinical question and use evidence-based research to address their questions. As library faculty regularly assist students with locating and evaluating sources at the reference desk and nursing faculty request library instruction sessions for their classes, we partnered with nursing faculty to assess the IL skills of EOLC students. Three nursing faculty members agreed to participate, providing PICO papers and advice on assessment materials.

This paper describes our creation and testing of a rubric to evaluate student IL skills as evidenced by PICO papers. Although we explored a few IL frameworks used with practicing nurses or nursing students [4–6], we ultimately chose the ACRL Framework because of the transferability of its skills to multiple disciplines and its place as the primary professional document for academic instruction librarians. Parallel key competencies of the American Nurses Association (ANA) Standards of Professional Nursing Practice [7] were then chosen and mapped onto to the six ACRL frames. Our goal was twofold: to better incorporate nursing faculty into the initiative and to offer students a means of assessing their capabilities as information literate health care professionals. Once the rubric was complete, we used it to score PICO papers, which provided us with valuable information regarding student IL skills and suggestions for further calibration of the rubric.

## METHODS

### Participants

Nursing professors provided fifty-two PICO papers written by students enrolled in three EOLC sections. The students were primarily seniors in college. Demographic information about the students was not collected, and names were redacted from the papers by the nursing faculty to protect student privacy. We applied for and received an exemption from formal review from the university’s institutional review board. The exemption was granted on the grounds that this study was considered a normal educational practice in an accepted educational environment.

### Materials

To create the rubric, we selected one knowledge practice for each of the six frames in the ACRL Framework that were most closely related to successful completion of the PICO assignment. The supplementary appendix provides the rubric. Next, we consulted the ANA standards, which detail the obligations that all registered nurses must fulfill, to identify parallel learning outcomes. We chose six standards that pertained to knowledge practices selected from the ACRL Framework: 1. Assessment, 3. Outcomes Identification, 4. Planning, 5. Implementation, 5D. Prescriptive Authority and Treatment, and 9. Evidence-Based Practice and Research. These standards were mapped onto each ACRL frame in the rubric. Three levels of student achievement were defined: beginning (1), developing (2), and exemplary (3). The nursing professors were sent a draft of the rubric to solicit their expert opinion; only minor edits were performed; and their feedback was used to finalize the rubric.

### Procedure

To achieve objective grading, we conducted practice scoring sessions with two randomly selected papers. Two investigators used the rubric to independently assess each paper and then met to discuss their scoring protocols and results. These two papers were then discarded from further analysis. Next, we independently scored the remaining fifty papers. After completion of scoring, we calculated descriptive statistics for each of the six rubric frames. To assess scoring consistency between the two raters, intra-class correlation coefficients (ICCs) were calculated to determine inter-rater reliability [8]. ICC values were calculated using SPSS software (model: two-way mixed, average measures; type: consistency).

## RESULTS

In general, student performance on the PICO papers varied between developing (2) and exemplary (3) (Table 1). Investigator 1 awarded students an exemplary score on all dimensions except for “Scholarship as Conversation,” which received a 2.5. Investigator 2 awarded students a developing score on every dimension but “Information Has Value.” The lowest median scores were given for the “Scholarship as Conversation” dimension, and the highest median scores were given for the “Information Has Value” dimension.

**Table 1:**
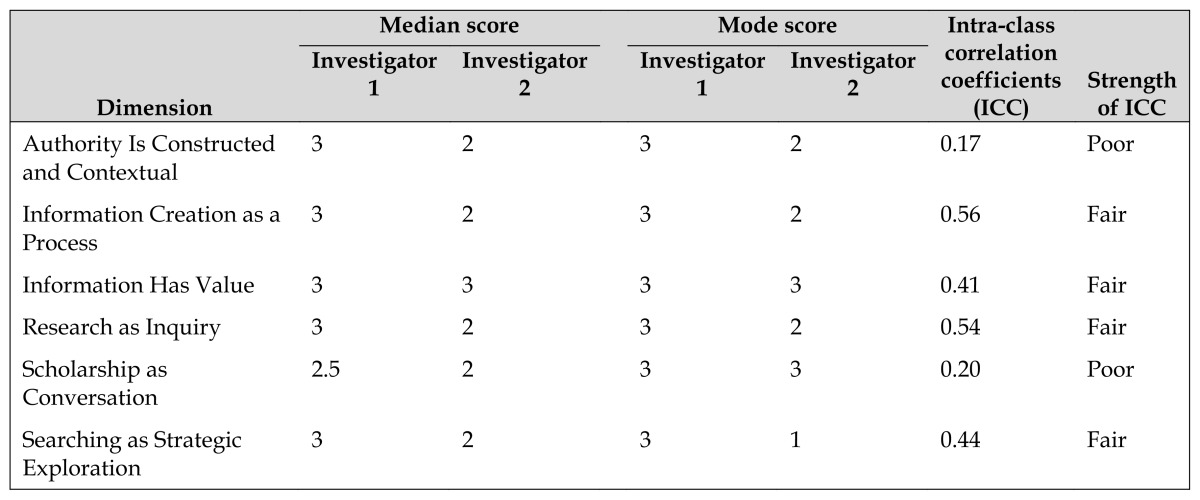
Student performance and inter-rater reliability

Dimension	Median score		Mode score		Intra-class correlation coefficients (ICC)	Strength of ICC
	
	Investigator 1	Investigator 2	Investigator 1	Investigator 2		
Authority Is Constructed and Contextual	3	2	3	2	0.17	Poor
Information Creation as a Process	3	2	3	2	0.56	Fair
Information Has Value	3	3	3	3	0.41	Fair
Research as Inquiry	3	2	3	2	0.54	Fair
Scholarship as Conversation	2.5	2	3	3	0.20	Poor
Searching as Strategic Exploration	3	2	3	1	0.44	Fair

However, examination of the scoring patterns of the two investigators revealed prominent differences in their assessment of student mastery of IL competencies (Table 1), with Investigator 1 awarding students higher median scores than Investigator 2 in five of the six frames. ICC values indicated “poor” or “fair” agreement [9] for all six frames.

## DISCUSSION

The objectives of this study were to create a rubric for evaluating IL competencies of nursing students and to apply the rubric to the assessment of a student research paper assignment. Our results show varying levels of student achievement among the six IL competencies as well as a need for greater calibration of the rubric due to low inter-rater reliability.

Holmes and Oakleaf describe steps that can be taken to norm rubrics to help ensure the validity and reliability of evaluating student skills across time and raters [9]. These steps include nominating a facilitator to lead the norming process, scoring the student artifacts with a draft rubric before norming, and collaboratively addressing divergent scores. While we piloted use of the rubric with two sample papers and made minor revisions to the rubric, our scoring consistency could have been improved by following Holmes and Oakleaf’s steps. In the future, other academic librarians who had been trained in IL instruction but were unaffiliated with the study could be invited to participate in the norming process. The creation of an instructional manual for the rubric would also be useful, as it could explain the scoring protocol for other investigators or educators who are interested in using this tool.

In terms of student achievement, both investigators agreed that the IL skill most evidenced by the students was “Information Has Value.” This dimension from the ACRL Framework pertains to citing and referencing, and the reference style format in question is that of the American Psychology Association (APA). The “exemplary” median scores awarded by both investigators may have resulted from a combination of students’ familiarity with APA style by their senior year and the ability of online databases to automatically generate references. By contrast, the investigators tended to award “developing” scores for “Scholarship as Conversation.” The poorer student achievement in this area suggested that these undergraduate students had not yet mastered the sophisticated scholarly communications skills of experts and advanced researchers. Scores on this dimension could be anticipated to rise as students enter graduate school or the nursing profession, where they would be immersed more fully into the nursing practice, language, and specialized body of knowledge.

The poor inter-rater reliability in rubric scores might be explained by differences in the background and experience of the two investigators. Whereas Investigator 1 was an instruction librarian with seven years of professional experience and a social sciences background, Investigator 2 was a health sciences librarian with fourteen years of professional experience. Therefore, the higher scores awarded by Investigator 1 might have been due to her being relatively new to the subject matter and assessment of nursing assignments. By contrast, the lower scores awarded by Investigator 2 might have been due to her greater familiarity and experience working with nursing students, leading her to hold these students to a higher standard. Another limitation of this project was the lack of student demographic information such as age, gender, and grade point average, which could have allowed the relationships between these variables and student achievement to be tested. In the future, therefore, nursing professors could be asked to release this information.

While most of previous literature has considered the ACRL Standards and Framework to be guiding documents for planning IL instruction [10, 11], we employed the Framework to construct a rubric to assess whether students achieved selected knowledge practices. Looking for evidence of IL skills achievement in end-of-program student artifacts may be an option for academic librarians in addition to using the Framework as a planning tool and/or using pre- and post-surveys of student learning.

In conclusion, the ACRL Framework can be used to guide the creation of valid and reliable assessment tools to measure student IL competencies. At present, there is little evidence of the existence of rubrics grounded in the Framework. The rubric employed in this study serves as an example of a flexible tool that librarians can combine with the standards of another profession to help students and instructors improve and translate important IL skills across disciplines and institutions.

## Supplemental File

Appendix Evaluation rubric using the Association of College and Research Libraries Framework and American Nurses Association Standards of Professional Nursing Practice
