# Supplementary material for: Mapping the Association of College and Research Libraries information literacy framework and nursing professional standards onto an assessment rubric
Source: J Med Libr Assoc. 2017 Apr;105(2):150–4. doi: 10.5195/jmla.2017.39 (PMC5370606; doi:10.5195/jmla.2017.39)
Supplement: Appendix [file jmla_apr17_willson_app.pdf]

JMLA Journal of the Medical Library Association

SC
Supplemental content to
J Med Libr Assoc. Apr;105(2): dx.doi.org/10.5195/jmla.2017.39
www.mlanet.org
© Willson, Angell 2017

Mapping the Association of College and Research Libraries information literacy framework and nursing professional standards onto an assessment rubric

Gloria Willson, MLIS, MPH; Katelyn Angell, MLIS, MA

APPENDIX

Evaluation rubric using the Association of College and Research Libraries Framework and American Nurses Association (ANA) Standards of Professional Nursing Practice

Association of College and Research Libraries Framework	American Nurses Association (ANA) Standards of Professional Nursing Practice	Beginning (1)	Developing (2)	Exemplary (3)
Authority Is Constructed and Contextual	5D. Prescribes evidence-based treatments, therapies, and procedures considering the health care consumer's comprehensive health care needs.	Seldom acknowledges development of own authoritative voice in nursing and seldom recognizes the responsibilities this entails, including seeking accuracy and reliability, respecting intellectual property, and participating in community of practice	Partially acknowledges development of own authoritative voice in nursing and partially recognizes the responsibilities this entails, including seeking accuracy and reliability, respecting intellectual property, and participating in community of practice	Fully acknowledges development of own authoritative voice in nursing and fully recognizes the responsibilities this entails, including seeking accuracy and reliability, respecting intellectual property, and participating in community of practice
Information Creation as a Process	9. Participates, as appropriate to education level and position, in the formulation of evidence-based practice through research	Seldom develops, in their own creation process, an understanding that their choices impact the purposes for which their information product will be used and the message it conveys	Partially develops, in their own creation process, an understanding that their choices impact the purposes for which their information product will be used and the message it conveys	Fully develops, in their own creation process, an understanding that their choices impact the purposes for which their information product will be used and the message it conveys

JMLA Journal of the Medical Library Association

Supplemental content to
J Med Libr Assoc. Apr;105(2): dx.doi.org/10.5195/jmla.2017.39
www.mlanet.org
© Willson, Angell 2017

Association of College and Research Libraries Framework	American Nurses Association (ANA) Standards of Professional Nursing Practice	Beginning (1)	Developing (2)	Exemplary (3)
Information Has Value	4. Integrates current scientific evidence, trends, and research	Seldom gives credit to the original ideas of others though proper attribution and citation	Partially gives credit to the original ideas of others though proper attribution and citation	Fully gives credit to the original ideas of others though proper attribution and citation
Research as Inquiry	1.Synthesizes available data, information, and knowledge relevant to the situation to identify patterns and variances	Seldom draws reasonable conclusions based on the analysis and interpretation of information	Partially draws reasonable conclusions based on the analysis and interpretation of information	Fully draws reasonable conclusions based on the analysis and interpretation of information
Scholarship as Conversation	5. Applies appropriate knowledge of major health problems and cultural diversity in implementing the plan of care	Seldom identifies the contribution that the selected articles make to disciplinary knowledge	Partially identifies the contribution that the selected articles make to disciplinary knowledge	Fully identifies the contribution that the selected articles make to disciplinary knowledge
Searching as Strategic Exploration	3. Considers associated risks, benefits, costs, current scientific evidence, expected trajectory of the condition, and clinical expertise when formulating expected outcomes	Seldom determines the initial scope of the assignment required to meet their information needs	Partially determines the initial scope of the assignment required to meet their information needs	Fully determines the initial scope of the assignment required to meet their information needs
